# Forensic microbiology reveals that *Neisseria animaloris* infections in harbour porpoises follow traumatic injuries by grey seals

**DOI:** 10.1038/s41598-019-50979-3

**Published:** 2019-10-11

**Authors:** Geoffrey Foster, Adrian M. Whatmore, Mark P. Dagleish, Henry Malnick, Maarten J. Gilbert, Lineke Begeman, Shaheed K. Macgregor, Nicholas J. Davison, Hendrik Jan Roest, Paul Jepson, Fiona Howie, Jakub Muchowski, Andrew C. Brownlow, Jaap A. Wagenaar, Marja J. L. Kik, Rob Deaville, Mariel T. I. ten Doeschate, Jason Barley, Laura Hunter, Lonneke L. IJsseldijk

**Affiliations:** 1 Scottish Marine Animal Stranding Scheme, SRUC Veterinary Services, An Lochran, 10 Inverness Campus, Inverness, IV2 5NA Scotland UK; 2 APHA Weybridge, Woodham Lane, Addlestone, Surrey KT15 3NB UK; 3 Moredun Research Institute, Pentlands Science Park, Bush Loan, Penicuik, Midlothian, EH26 0PZ Scotland UK; 4 Laboratory of Health Care Associated Infection, Public Health England, Colindale, London NW9 5EQ UK; 5 Department of Infectious Diseases and Immunology, Faculty of Veterinary Medicine, Utrecht University, Yalelaan 1, 3584CL Utrecht, The Netherlands; 6 Department of Viroscience, Erasmus University Medical Centre, Wytemaweg 80, 3015 CN Rotterdam, The Netherlands; 7 Cetacean Stranding Investigation Programme, Institute of Zoology, Regent’s Park, London, NW1 4RY UK; 8 Department of Bacteriology and Epidemiology, Wageningen Bioveterinary Research, Houtribweg 39, 8221 RA Lelystad, The Netherlands; 9 SRUC Veterinary Services, Bush Estate, Penicuik, Midlothian, EH26 OQE Scotland UK; 10 Faculty of Veterinary Medicine, Department of Pathobiology, Utrecht University, Yalelaan 1, 3584CL Utrecht, The Netherlands; 11 Present Address: Veterinary Sciences Division, Agri-Food and Biosciences Research Institute, Stoney Road, Stormont, Belfast, BT4 3SD Northern Ireland UK

**Keywords:** Conservation biology, Population dynamics, Bacteriology, Marine biology, Bacterial infection

## Abstract

*Neisseria animaloris* is considered to be a commensal of the canine and feline oral cavities. It is able to cause systemic infections in animals as well as humans, usually after a biting trauma has occurred. We recovered *N*. *animaloris* from chronically inflamed bite wounds on pectoral fins and tailstocks, from lungs and other internal organs of eight harbour porpoises. Gross and histopathological evidence suggest that fatal disseminated *N*. *animaloris* infections had occurred due to traumatic injury from grey seals. We therefore conclude that these porpoises survived a grey seal predatory attack, with the bite lesions representing the subsequent portal of entry for bacteria to infect the animals causing abscesses in multiple tissues, and eventually death. We demonstrate that forensic microbiology provides a useful tool for linking a perpetrator to its victim. Moreover, *N*. *animaloris* should be added to the list of potential zoonotic bacteria following interactions with seals, as the finding of systemic transfer to the lungs and other tissues of the harbour porpoises may suggest a potential to do likewise in humans.

## Introduction

The mucosal surfaces of the oropharynx are considered the principal habitats of *Neisseria* spp. recovered from humans and a number of domestic and experimental animals^1^. Reports from wild animals are less common, however, *Neisseria zalophili* was described recently from the oral cavity of wild California sea lions^2^, suggesting that pinnipeds may similarly harbour members of the *Neisseria* genus.

*Neisseria animaloris* was described for a group of organisms that had been placed originally in the Centers for Disease Control (CDC) group eugonic fermenter (EF)-4a^3^. The organism is considered to be a commensal of the oral cavity of cats and dogs and can cause systemic infections in both humans and animals^4^. Two biotypes, EF-4a and EF-4b, were described initially on the basis of arginine hydrolysis^5^ and were further distinguished according to cellular fatty acid content^6^ and whole-cell protein analysis^7^. The arginine positive strains (EF-4a) were subsequently described as *N*. *animaloris*^3^.

Here, we report the recovery of *N*. *animaloris* from eight harbour porpoises (*Phocoena phocoena*) which stranded in four countries of Northern Europe: Scotland, England, the Netherlands and Belgium. Gross and histopathological evidence suggests these infections occurred following traumatic injury from a predator and the wounds fitted with those described to be inflicted upon porpoises by grey seals (*Halichoerus grypus*)^8^.

## Results

### Gross and histopathology

**Figure 1:**
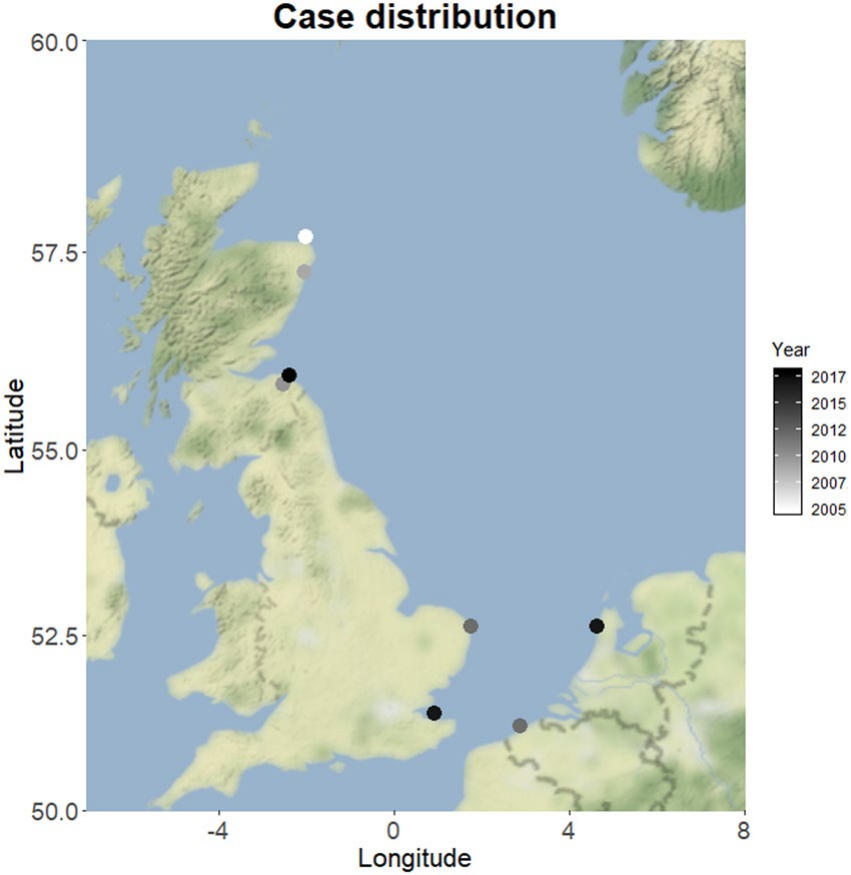
Case distribution throughout the area, with temporal colour scaling, where white is the oldest case (2005) and black is the most recent case (2018).

The harbour porpoises were six adult females and two juveniles; one male and one female. All animals were in moderate to poor nutritional condition at the time of stranding. Six animals were found dead but all showed minimal decomposition, although one animal had severe scavenger damage. Two animals had live stranded and were subsequently euthanized on welfare grounds due to the severity of their condition (Table S1). Stranding locations varied temporally and spatially (Fig. 1).

**Figure 2:**
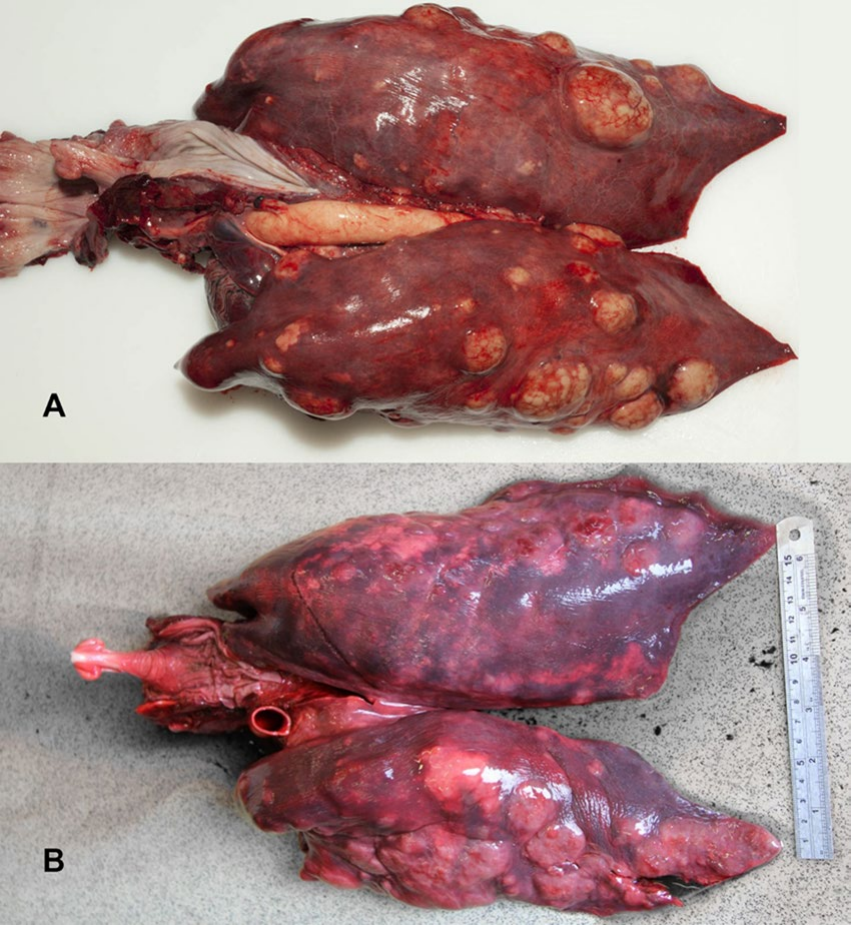
Respiratory tract of UT692 (**A**) and M175/18 (**B**) presenting with strong similarity in the macroscopic morphology of the lung lesions: multiple well demarcated, 5–60 mm yellow abscesses bulging from the surface. Photo’s: Multimedia, UU (**A**) and SMASS (**B**).

**Figure 3:**
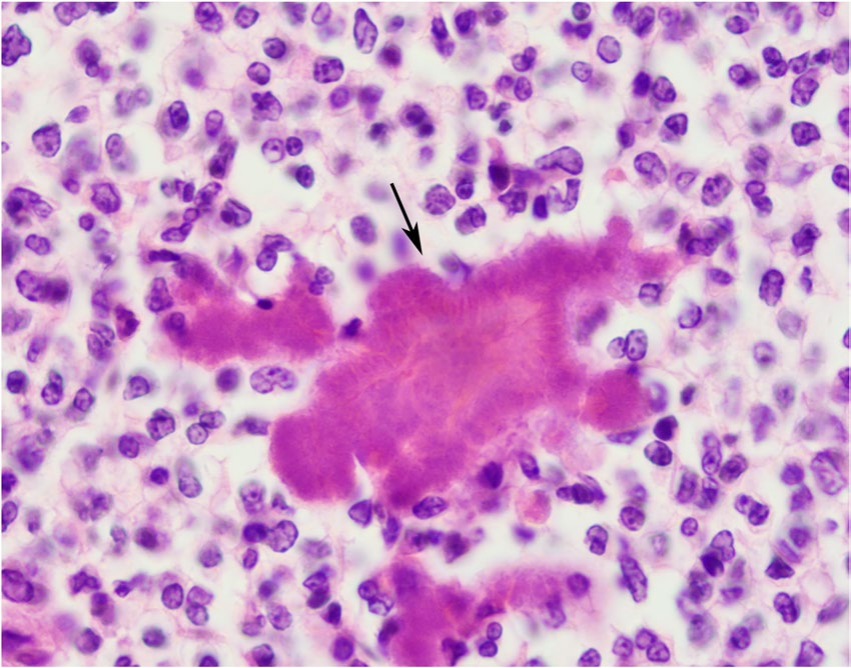
Lung of UT1576, HE stain, 100x magnification; typical example of a *Neisseria* abscess, with large numbers of degenerate neutrophils, admixed with typical aggregates of bacteria, which are surrounded by eosinophilic, acellular material that form radiating clubs (Splendore-Hoeppli material indicated by arrow).

The ultimate cause of death for all harbour porpoises was a disseminated bacterial infection causing abscesses in multiple tissues. All harbour porpoises where external assessment was possible (n = 7) presented with abscesses and inflamed lacerations in the skin at specific sites: pectoral fins (n = 3), tailstock (n = 3) and in one case on a pectoral fin and the tailstock. In addition, abscesses were present in the lungs in all cases (n = 8), in one or several lymph nodes in six of eight cases and in the spleen of one case. Abscesses were characterized as well circumscribed, large (up to 7 cm in diameter) masses, with fibrous walls containing caseous exudate on cut section (Fig. 2). Lung abscesses were randomly distributed throughout the lung parenchyma suggesting haematogenous spread. Lymph node abscesses occurred, varying per case, in pulmonary lymph nodes, prescapular lymph nodes and mesenteric lymph nodes. Histologically the abscesses were similar and characterized by well-demarcated masses with fibrous capsules and centrally large numbers of neutrophils and macrophages (pyogranuloma), few erythrocytes and intralesional aggregates of bacteria. Bacteria were non-filamentous, non-branching and surrounded by eosinophilic, acellular material that formed radiating clubs (Splendore-Hoeppli material) (Fig. 3).

Other pathological lesions which can be characteristic of bacterial sepsis and therefore might have been related to the *Neisseria* infection, were noted, including multifocal acute hepatic necrosis (n = 2), haemorrhages in brain and adrenal glands (n = 1); and mild acute cortical necrosis in the adrenal glands (n = 1). Two livers exhibited fat accumulation (lipidosis), a change associated with an unknown period of anorexia prior to death.

Findings related to a helminth parasite burden, considered unrelated to the *Neisseria* infection, or the ultimate cause of death, were noted in lungs, liver and auditory sinuses (Table S2). Parasitic infections are common in free-ranging harbour porpoises and their clinical relevance is uncertain^9^.

**Figure 4:**
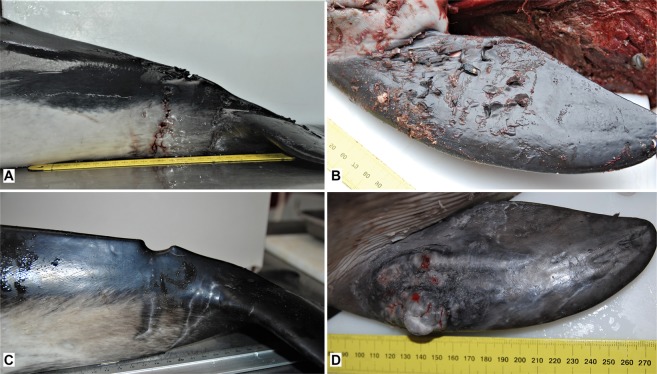
Acute grey seal bite wound on tailstock (**A**, case ref no. UT1004) and pectoral fin (**B**, case ref no. UT1007) from cases for which DNA analysis proved that lesions were inflicted by grey seals in a previous study^20^. Healed tailstock lesions (**C**, case no. UT1576) and an abscess on the pectoral fin (**D**, case ref no. UT692) show morphological similarities to the acute cases (**A**,**B**), although lesions presented in (**C**,**D**) are chronic.

Macroscopic photos and/or detailed external descriptions were available from all cases for the evaluation of external evidence of lesions consistent with bite marks induced by grey seals, based on comparison with a previous study^8^. Lesions were bilaterally or ventrodorsally, close to symmetrical, present on head, pectoral fins and/or tailstocks. Though these lesions were chronic, based on tissue response, they were otherwise consistent with lesions induced by grey seals based on location and morphology of the lesions (Fig. 4). For one case (M21/09) the carcass had been scavenged so severely that presence or absence of external lesions could not be assessed reliably. For this case it remains uncertain if the *Neisseria* infection could have been related to seal bites.

### Microbiology

A Gram negative coccal-shaped organism which formed non-haemolytic, yellow, sticky colonies on Columbia sheep blood agar (CSBA) was recovered from all abscesses cultured. A preference for additional CO_2_ was demonstrated. The isolates were sensitive to 1 unit of penicillin, but did not produce elongated cells at the edge of growth closest to the disc, indicating that they were Gram negative cocci. Growth was obtained anaerobically and at 25 °C, but not at 42 °C. Growth on MacConkey agar without salt varied between isolates. Isolates from 6 animals (M29/05, M21/09, M78/10, SW12/463, UT1576 and M175/18) were tested in a panel of biochemical tests and results were consistent between each other and with those described for *N*. *animaloris*. Tests were positive for catalase and oxidase, but negative for indole. Nitrates were reduced to nitrite with API 20E and 20NE. Acid production was produced from glucose with API 50CH, but was negative with API20E and 20NE and produced varied results with glucose DIATABS. The only other positive carbohydrate detected was ribose in the API 50CH system. Arginine dihydrolase was positive with both API 20E and DIATABS, while all other tests in the API 20E, 20NE and DIATABS were negative. Using API ZYM, the only positive tests were weak reactions for esterase C4, esterase C8, leucine arylamidase and acid phosphatase. *Neisseria animaloris* is not included in the API databases, however, results, where available, were similar to those obtained by traditional methods in the species description^3^. API ZYM reactions matched those for the type strain of *N*. *animaloris* available from the website of the Culture Collection of the University of Goteburg (CCUG 52597T), with the exception that the type strain had a weak reaction for alkaline phosphatase (http://www.ccug.se).

Whole-cell fatty acid analysis was performed on the first three isolates (M29/05, M21/09, M78/10), which identified them as *N*. *animaloris*, the results being consistent with those of Vandamme *et al*.^3^. Ranges of percentage cellular fatty acid composition of the first 3 isolates were C_12: 0_, 8–8.4, C_12: 0 3-OH_, 4.6–5, C_14: 0_, 7.2–10, C_16:1 w5c_, 1.2–1.6, C_12: 0_, 8–8.4 C_12: 0 3-OH_, 4.6–5, C_14: 0_, 7.2–10, C_16:1 w5c_, 1.2–1.6, C_16:1 w7c_, 25.3–30.5, C_16: 0_, 21–24, C_18: 1ω7c_, 16.5–20.3, C_18:0_, all 3 trace, summed feature 2 (C_14:0 3-OH_, C_16:1 iso_, an unidentified fatty acid with equivalent chain-length value of 10.928 or C_12_: ALDE, or any combination of these fatty acids) 3.5–5.

**Figure 5:**
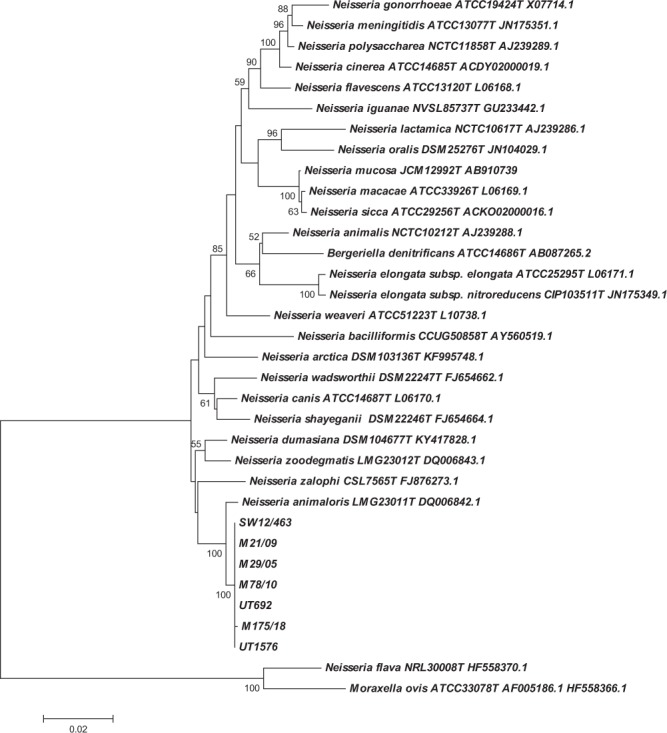
Phylogenetic analysis of the porpoise strains in comparison with type strains of members of the genus *Neisseria* inferred from 16S rRNA sequence. Labelling shows the strain name and the corresponding accession number for the sequence used in this comparison. Numbers at nodes correspond to proportions of 100 resamplings that support the topology shown with only values >50% indicated. Bar = 0.02 substitutions per nucleotide position.

**Figure 6:**
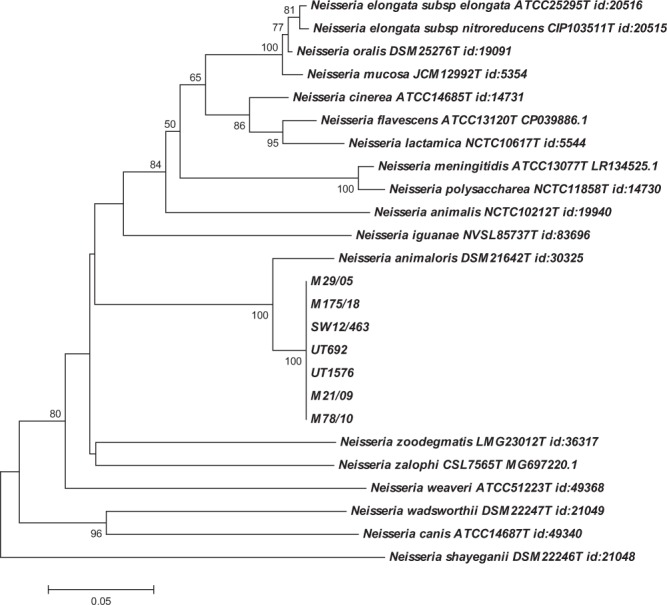
Phylogenetic analysis of the porpoise strains in comparison with type strains of members of the genus *Neisseria* inferred from *rplF* sequence. Labelling shows the strain name and the corresponding accession number for the sequence used in this comparison either from NCBI or the PubMLST database (see Methods). Numbers at nodes correspond to proportions of 100 resamplings that support the topology shown with only values >50% indicated. Bar = 0.05 substitutions per nucleotide position.

Sequencing of the 16S rRNA gene was carried out for seven of the eight isolates with a closest match to *N*. *animaloris* on BLAST analysis. Over a 1390 bp contig available for all seven isolates either 8 (n = 6), or 9 (n = 1), nucleotide mismatches were apparent compared to the type strain of *N*. *animaloris* LMG23011^T^, equivalent to 99.35–99.42% identity. Phylogenetic analysis, in comparison with type strains of all recognised *Neisseria* spp., where equivalent length 16S rRNA sequence was available, (equating to a 1348 bp contig for the isolates described here), confirmed the identity of the isolates as most closely related to *N*. *animaloris* (Fig. 5). The porpoise isolates do appear to represent a clonal complex somewhat distinct from the species type strain, however there is a paucity of information on potential 16S rRNA gene diversity with this species. Further, while distinct from the *N*. *animaloris* type strain, even the most diverse sequence shares >99.3% nucleotide identity with it. While there are no strict criteria for species delineation based on 16S rRNA sequence this is consistent with the 98.7–99% threshold often quoted for confirmation of species identity^10,11^. As further evidence of the relationship of these isolates to *N*. *animaloris* sequencing of a fragment of *rplF* was performed. This gene encodes the 50S ribosomal protein L6 and has been identified as a suitable target for differentiation within *Neisseria* spp. as phylogenies constructed from this fragment are consistent with phylogenies constructed from concatenated sequences of 53 whole-ribosomal protein genes^12^. Over a 414 bp fragment, identical in all seven strains examined, sequences shared 18 nucleotide mismatches (95% identity) with the *N*. *animaloris* type strain sequence. Phylogenetic analysis with available equivalent sequences from type strains showed a similar relationship to that based on 16 S rRNA with the porpoise isolates clearly most closely related to *N*. *animaloris*, but again representing a clonal complex somewhat distinct from the type strain (Fig. 6). Further, isolates of all cases were identified as *N*. *animaloris* with scores ≥2.0 using matrix-assisted laser desorption-ionisation time-of-flight mass spectrometry (MALDI-TOF MS), a result considered accurate to the species level according to the database supplied by the manufacturer.

*Neisseria animaloris* was the dominant isolate, often in pure growth, from lung abscesses of all cases (n = 8). Additionally, *N*. *animaloris* was cultured from skin abscesses on pectoral fins (n = 3), tailstock (n = 1), inguinal area (n = 1) and shoulder (n = 1). Other recovery sites for *N*. *animaloris* and other isolates recovered are listed in Table S4. Additional isolates included *Brucella ceti* (M175/18) and a monophasic strain of *Salmonella enterica* with the antigenic formula, 4, 12:a:-, which is host-adapted to porpoises (M29/05 and M21/09) (Table S4), but deemed unrelated to the ultimate cause of death of these cases.

## Discussion

Here we report the identification of *N*. *animaloris* recovered from eight harbour porpoises. We could not find any previous reports of the isolation of *N*. *animaloris* from any marine mammal species. Furthermore, *N*. *animaloris* has not been recovered from any of the other more than 800 harbour porpoises examined bacteriologically in the UK over the last thirty years, some of which had abscesses without evidence of grey seal bites (unpublished data). In all eight cases, the organism was recovered in sole or dominant growth from lung abscesses, with additional recovery from other tissues including pectoral fin and tailstock abscesses. The cases for which it was possible to assess (7/8), all demonstrated external lesions that were consistent with grey seal related injuries.

There are a number of other species known to interact with harbour porpoises, but those can all be excluded as potential inflictors of the bite lesions as described on the eight cases. In the North Sea, aggressive behaviour towards harbour porpoises by white-beaked dolphins (*Lagenorhynchus albirostris*)^13^ and bottlenose dolphins (*Tursiops truncatus*)^14,15^ have been well-described. Porpoises that died following aggressive interactions with delphinids present clearly defined rake marks, which were not present on any of the cases from which *N*. *animaloris* was cultured. Killer whales (*Orcinus orca*) and large sharks do not inhabit the North Sea and only very rarely occur in adjacent waters. Finally, other animals known to interact with stranded porpoises are scavengers, like red foxes (*Vulpes vulpes*) or birds^16^, but scavenging does not explain the presence of *N*. *animaloris* in chronic lesions and internal organs of the eight cases presented here. The only other large marine animal in the North Sea is the harbour seal (*Phoca vitulina*), but seeing their significantly smaller body size and inter-teeth distance, it is less likely that harbour seals are responsible for the bite wounds on harbour porpoises and interactions between harbour seals and porpoises have to date not been described. This leaves the abundant grey seal as the most likely perpetrator of the infected wounds.

*Neisseria animaloris* is considered to be a constituent of the normal flora of the canine and feline oral cavity and has also been the cause of zoonotic infections following bites from these two domestic species^1^. It is intriguing to consider that the route of infection in the harbour porpoises is similar, following seal bites. *Neisseria animaloris* has not been reported from grey seals previously and its culture from the mouth of these animals, where it may reside, possibly in low numbers in a highly populated and diverse microbial environment, may represent a significant challenge. Microbiome investigation of the oral cavity of nine grey seals, however, detected a *Neisseria* 16S sequence which showed 100% sequence ID with our isolates (based on a 437 bp sequence) in the oral cavity of four animals, indicating that at least some grey seals appear to carry *N*. *animaloris* (publication in progress). In contrast, this specific *Neisseria* 16S sequence was absent from the oral cavities of harbour seals (n = 8) and harbour porpoises (n = 6). The sequence showed 99% sequence ID with *N*. *animaloris* obtained from terrestrial carnivores and humans, including the *N*. *animaloris* type strain.

Grey seal predation on harbour porpoises was first suggested by Haelters *et al*.^17^ who matched lesions present on mutilated porpoises to the inter-teeth-distance of grey seals. This theory was proven through the documentation of field observations of (fatal) interactions^18,19^ and retrieval of grey seal DNA from bite marks on mutilated harbour porpoises^20,21^. Characteristics of bite marks on harbour porpoises were retrospectively assessed evaluating post-mortem photos of harbour porpoises in the Netherlands and it was concluded that predation by grey seals is one of the main causes of death^8^. It is known that not all grey seal attacks are (directly) fatal^22^. In the study of Leopold *et al*.^8^, 46 ‘possible escaped harbour porpoises’ (6% of the studied sample) lacked large mutilations, but had bite marks with associated inflammation. The observed and likely grey seal related injuries on the harbour porpoises in this study and the concurrent retrieval *of N*. *animaloris* from these cases, in addition to the absence of *N*. *animaloris* recovery from harbour porpoise lesions without evidence of grey seal assault, lead us to conclude that all these porpoises survived a grey seal attack. The bite lesions represent the subsequent portal of entry for bacteria to infect the animals, causing abscesses, as well as multiple organ infection, and eventually death.

The characterization and quantification of lesions on stranded marine mammals is vital, in order to differentiate between causes, with the aim of providing useful metrics to distinguish between lesions induced by direct human related causes of death, such as fisheries bycatch and from natural causes of death, such as inter-species interaction. There is an increasing need to assess any population level effects of interactions between sympatric species, which is of particular relevance for porpoise population assessments, given the observed exponential increase in grey seal numbers in many areas of the North Sea^23^. The identification of a predator through forensic DNA has successfully been used in terrestrial settings^24,25^ as well as in the identification and differentiation between grey seal predation and fox scavenging on marine mammals^16,20,26^. In this study, we have demonstrated a further potentially powerful tool for the identification of a perpetrator: forensic microbiology through identification of *N*. *animaloris* in eight harbour porpoises and linkage to associated bite wounds from grey seals. Further support is provided by the finding of *N*. *animaloris* in grey seal microbiome studies whereby there was a 100% match with the porpoise isolates and a 99% match with the terrestrial strain NCTC 12227 based on 437 bp. Approaches to use bacteria in forensic science have been reported previously, with key examples in the identification of individuals through individual-specific skin bacterial communities^27^.

Finally, our findings add to concerns with respect to zoonotic infections of humans following seal bites and the handling of stranded seals and harbour porpoises. The association of *N*. *animaloris* to human infections following dog and cat bites suggests that seal isolates may have the potential to act similarly following infection from seals or porpoises. Therefore, *N*. *animaloris* should be added to the list of potential zoonotic organisms when handling marine mammals. *Neisseria animaloris* is absent from the databases of most commercial phenotypic identification kit suppliers, which potentially hinders its recognition from clinical samples. Diagnostic laboratories are increasingly turning away from commercial phenotypic biochemical kits, however, with MALDI-TOF MS becoming a preferred choice for first line identification of bacterial isolates. As *N*. *animaloris* is already included in the Bruker MALDI-TOF database and with 16 S rRNA sequencing being a further option, the recognition of *N*. *animaloris*, whether from humans, dogs, cats, seals or other animals, should be more likely in the future.

## Materials and Methods

### Specimens

The harbour porpoises reported here were all free-ranging animals that were found dead or died due to, or shortly after stranding. They were examined by different institutes depending on the geographic location of stranding: four harbour porpoises were examined in Scotland under the Scottish Marine Animals Strandings Scheme (SMASS); two animals from England under the Cetacean Stranding Investigation Program (CSIP) and two in the Netherlands at the faculty of Veterinary Medicine of Utrecht University (UU).

### Gross- and histopathology

Post-mortem examinations and subsequent tissue collections were carried out following a recognised protocol for small cetacean necropsies^28^. Tissues available for histologic review varied from animal to animal, but included: skin (with any lesions), muscle, prescapular lymph nodes, lung and associated lymph nodes, heart, liver, adrenals, kidney, oesophagus, stomachs, spleen, pancreas, intestine and associated lymph nodes, brain and spinal cord. Tissues were fixed in 10% neutral buffered formalin, processed routinely through graded alcohols, embedded in paraffin-wax, sectioned at 4–7 µm, stained with haematoxylin and eosin (HE) and examined by light microscopy. Additional stains were performed when appropriate, including the Periodic acid Schiff stain to detect fungi. For the cases in which photos of the external lesions were available the photos were evaluated for the presence of lesions characteristic of grey seal attacks based on methods previously described^8^. If photos were not available the macroscopic descriptions of the lesions were used to judge if wounds had been caused by seal bites.

### Microbiology

Samples of lung and abscesses were collected and processed for microbiology from all harbour porpoises included in this study. Other tissues available for microbiology varied from animal to animal, but included liver, spleen, kidney, brain, mesenteric lymph node and intestine. Cultures were made on Columbia sheep blood agar (CSBA) (Oxoid, Basingstoke, UK), incubated at 37 °C in air plus 5% CO_2_ and MacConkey agar without salt (Oxoid), incubated aerobically at the same temperature. For cases processed at SMASS, selective cultures were set up for *Brucella* spp. as described previously^29^ and anaerobic cultures were made on fastidious anaerobe agar with horse blood and on the same medium with nalidixic acid and vancomycin added (Oxoid). Further characterisation of *N*. *animaloris* isolates included API 20E, 20NE, 50CH and ZYM systems (BioMerieux, Basingstoke, UK), according to the manufacturer’s instructions and with DIATABS (Rosco Diagnostica, Taastrup, Denmark) for the following tests: Voges-Proskauer, urease, aesculin hydrolysis, arginine dihydrolase, lysine decarboxylase, ornithine decarboxylase, dulcitol, glucose, lactose, maltose, mannitol, mannose, sorbitol, sucrose, trehalose, xylose, α-fucosidase, α-galactosidase, β-galactosidase, α-glucosidase, β-glucosidase and β-xylosidase.

Whole-cell fatty acid analysis was carried out to compare three isolates (cases M29/05, M21/09, M78/10) with those described for *N*. *animaloris* previously^3^. For cellular fatty acid (CFA) analysis, cultures were grown on trypticase soy agar (TSA; Becton Dickinson Co., Oxford, UK) at 30 °C aerobically for 24 hours. The CFAs were extracted and analysed by gas chromatography (MIDI Sherlock, Newark, DE) using the MIDI system’s rapid TSBA database. MALDI-TOF MS was performed on the Bruker MALDI Biotyper. Sequencing of the 16S rRNA gene was performed, essentially as described previously^30^. Phylogenetic comparison of 16S rRNA sequences was carried out with MEGA5.2 using the neighbour-joining approach with the pairwise deletion option following CLUSTAL alignment of sequences trimmed to a 1348 bp consensus contig (=1344 bp of *N*. *animaloris* sequences). This analysis included all available near full length 16S rRNA sequences from *Neisseria* type strains based on consultation of Jean Euzeby’s *List of Bacterial Names with Standing in Nomenclature* (http://www.bacterio.cict.fr/index.html) with the addition of the recently described *Neissera zalophi*^2^. Sequences have been submitted to GenBank and assigned accession numbers MK441685 through MK441690. Amplification and sequencing of a fragment of *rplF* was performed as described previously^12^. Phylogenetic comparison of sequences was carried out with MEGA5.2 using the neighbour-joining approach with the pairwise deletion option following CLUSTAL alignment of sequences trimmed to a 414 bp consensus contig. Sequences have been submitted to GenBank and assigned accession numbers MN242377 through MN242383. Type strain alleles of *rplF* were obtained from either NCBI or the PubMLST database (denoted with id nr in Fig. 6) at https://pubmlst.org/neisseria/.

## Supplementary information

Supplementary files

## Data Availability

All data generated or analysed during this study are included in this published article (and its Supplementary Information Files).

## References

[1] Tøne, T. Genus 1. Neisseria Trevisan 1885, 105AL. In: *Bergey’s Manual of Systematic Bacteriology Volume 2, the Proteobacteria Part C the alpha, beta, delta and epsilonproteobacteria* pp. 777–798. Edited by Brenner, D. J., Krieg, N. R. & Staley, J. T. Springer (2005).

[2] Volokhov DV (2018). *Neisseria zalophili* sp. nov., isolated from oral cavity of Californian sea lions (*Zalophilus californianus*. Arch. Microbiol..

[3] Vandamme P, Holmes B, Bercovier H, Coenye T (2006). Classification of Centers for Disease Control group eugonic fermenter (EF)-4a and EF-4b as *Neisseria animaloris* sp. nov. and *Neisseria zoodegmatis* sp. nov. respectively. Int. J. Syst. Evol. Microbiol..

[4] Ganière JP, Escande F, André-Fontaine G, Larrat M, Filloneau C (1995). Characterisation of group EF-4 bacteria from the oral cavity of dogs. Vet. Microbiol..

[5] Holmes, B. & Ahmed, M. S. Group EF-4: a Pasteurella-like organism. In: Haemophilus, Pasteurella and Actinobacillus, pp. 161–174. Edited by Kilian, M., Frederiksen, W. & Biberstein, E. L. London: Academic Press (1981).

[6] Dees SB, Powell J, Moss CW, Hollis DG, Weaver RE (1981). Cellular fatty acid composition of organisms frequently associated with human infections from dog bites: *Pasteurella multocida* and groups of EF-4, IIj, M-5, and DF-2. J. Clin. Microbiol..

[7] Holmes B, Costas M, Wood CA (1990). Numerical analysis of electrophoretic patterns of group EF-4 bacteria, predominantly from dog-bite wounds of humans. J. Appl. Microbiol..

[8] Leopold MF (2015). Exposing the grey seal as a major predator of harbour porpoises. Proc. R. Soc. Lond. B..

[9] Ten Doeschate MT (2017). Quantifying parasite presence in relation to biological parameters of harbour porpoises *Phocoena phocoena* stranded on the Dutch coast. Dis. Aquat. Organ..

[10] Stackebrandt E, Goebel BM (1994). Taxonomic note: a place for DNA-DNA reassociation and 16S rRNA sequence analysis in the present species definition in bacteriology. Int. J. Syst. Bacteriol..

[11] Drancourt M (2000). 16S ribosomal DNA sequence analysis of a large collection of environmental and clinical unidentifiable bacterial isolates. J. Clin. Microbiol..

[12] Bennett JS, Watkins ER, Jolley KA, Harrison OB, Maiden MC (2014). Identifying Neisseria species by use of the 50S ribosomal protein L6 (rplF) gene. J. Clin. Microbiol..

[13] Haelters J, Everaarts E (2011). Two cases of physical interaction between white-beaked dolphins *Lagenorhynchus albirostris* and juvenile harbour porpoises *Phocoena phocoena* in the southern North Sea. Aquat. Mamm..

[14] Ross HM, Wilson B (1996). Violent interactions between bottlenose dolphins and harbour porpoises. Proc. R. Soc. Lond. B..

[15] Patterson IAP (1998). Evidence for infanticide in bottlenose dolphins: An explanation for violent interactions with harbour porpoises?. Proc. R. Soc. Lond. B..

[16] Haelters J (2016). A suspected scavenging event by red foxes (*Vulpes vulpes*) on a live, stranded harbour porpoise (*Phocoena phocoena*). Aquat. Mamm..

[17] Haelters J, Kerckhof F, Jauniaux T, Degraer S (2012). The grey seal (*Halichoerus grypus*) as a predator of harbour porpoises (*Phocoena phocoena*)?. Aquat. Mamm..

[18] Bouveroux T, Kiszka JJ, Heithaus MR, Jauniaux T, Pezeril S (2014). Direct evidence for gray seal (*Halichoerus grypus*) predation and scavenging on harbor porpoises (*Phocoena phocoena*). Mar. Mam. Sci..

[19] Stringell T (2015). Predation of harbour porpoises (*Phocoena phocoena*) by grey seals (*Halichoerus grypus*) in Wales. Aquat. Mamm..

[20] van Bleijswijk JDL (2014). Detection of grey seal *Halichoerus grypus* DNA in attack wounds on stranded harbour porpoises *Phocoena phocoena*. Mar. Ecol. Prog. Ser..

[21] Jauniaux T (2014). Bite injuries of grey seals (*Halichoerus grypus*) on harbour porpoises (*Phocoena phocoena*). PloS One.

[22] Podt A, IJsseldijk LL (2017). Grey seal attacks on harbour porpoises in the Eastern Scheldt: Cases of survival and mortality. Lutra.

[23] Special Committee on seals (SCOS). Scientific advice on matters related to the management of seal populations: St Andrews: Sea Mammal Research Unit, SCOS main advice, http://www.smru.st-andrews.ac.uk/files/2018/01/SCOS-2017.pdf (2017).

[24] Williams CL, Blejwas K, Johnston JJ, Jaeger MM (2003). A coyote in sheep’s clothing: predator identification from saliva. Wildl. Soc. Bull..

[25] Blejwas KM, Williams CL, Shin GT, McCullough DR, Jaeger MM (2006). Salivary DNA evidence convicts breeding male coyotes of killing sheep. J. Wildl. Manag..

[26] Heers T (2018). Loop-mediated isothermal amplification (LAMP) as a confirmatory and rapid DNA detection method for grey seal (Halichoerus grypus) predation on harbour porpoises (Phocoena phocoena). J. Sea Res..

[27] Fierer N (2010). Forensic identification using skin bacterial communities. Proc. Natl. Acad. Sci..

[28] Kuiken, T. & Hartmann, M. G. Dissection techniques and tissue sampling. Proceedings of the first ECS workshop on cetacean pathology, Leiden, The Netherlands, September 13–14, 1991. pp 1–39 (1993).

[29] Foster G (2002). A review of *Brucella* sp. Infection of sea mammals with particular emphasis on isolates from Scotland. Vet. Microbiol..

[30] Hunt B, Bidewell C, Koylass MS, Whatmore AM (2013). A novel taxon within the genus Actinobacillus isolated from alpaca (Vicugna pacos) in the United Kingdom. Vet Microbiol..

