# Supplementary material for: Forensic microbiology reveals that Neisseria animaloris infections in harbour porpoises follow traumatic injuries by grey seals
Source: Sci Rep. 2019 Oct 11;9:14338. doi: 10.1038/s41598-019-50979-3 (PMC6789040; doi:10.1038/s41598-019-50979-3)
Supplement: Supplementary file 1 — Supplementary files [file 41598_2019_50979_MOESM1_ESM.docx]

**Forensic microbiology reveals that *Neisseria animaloris* infections in harbour porpoises follow traumatic injuries by grey seals**

Geoffrey Foster,^1^* Adrian M. Whatmore,^2^ Mark P. Dagleish,^3^ Henry Malnick,^4^ Maarten J. Gilbert,^5^ Lineke Begeman,^6^ Shaheed K. Macgregor,^7^ Nicholas J. Davison,^1^ Hendrik Jan Roest,^8^ Paul Jepson,^7^ Fiona Howie,^9^ Jakub Muchowski,^2^ Andrew C. Brownlow,^1^ Jaap A. Wagenaar,^5,8^ Marja J. L. Kik,^10^ Rob Deaville,^7^ Mariel T. I. ten Doeschate,^1^ Jason Barley,^1,11^ Laura Hunter,^1^ and Lonneke L. IJsseldijk,^10^*

^1^ Scottish Marine Animal Stranding Scheme, SRUC Veterinary Services, Drummondhill, Stratherrick Road, Inverness IV2 4JZ, Scotland, UK

^2^ APHA Weybridge, Woodham Lane, Addlestone, Surrey KT15 3NB, UK

^3^ Moredun Research Institute, Pentlands Science Park, Bush Loan, Penicuik, Midlothian EH26 0PZ, Scotland, UK

^4^ Laboratory of Health Care Associated Infection, Public Health England, Colindale, London NW9 5EQ, UK

^5^ Department of Infectious Diseases and Immunology, Faculty of Veterinary Medicine, Utrecht University, Yalelaan 1, 3584CL, Utrecht, The Netherlands

^6^ Department of Viroscience, Erasmus University Medical Centre, Wytemaweg 80, 3015 CN, Rotterdam, The Netherlands

^7^ Cetacean Stranding Investigation Programme, Institute of Zoology, Regent’s Park, London NW1 4RY, UK

^8^ Department of Bacteriology and Epidemiology, Wageningen Bioveterinary Research, Houtribweg 39, 8221 RA Lelystad, The Netherlands

^9^ SRUC Veterinary Services, Bush Estate, Penicuik, Midlothian EH26 OQE, Scotland, UK

^10^Faculty of Veterinary Medicine, Department of Pathobiology, Utrecht University, Yalelaan 1, 3584CL, Utrecht, The Netherlands

^11^Current address: Veterinary Sciences Division, Agri-Food and Biosciences Research Institute, Stoney Road, Stormont, Belfast BT4 3SD, Northern Ireland, UK

*Corresponding authors: [Geoffrey.Foster@sac.co.uk](mailto:Geoffrey.Foster@sac.co.uk) and [L.L.IJsseldijk@uu.nl](mailto:L.L.IJsseldijk@uu.nl)

**Table S1** Life history and strandings details of the 8 harbour porpoise cases

| **Case ID** | **Strandings month and year** | **Strandings location** | **Age class and sex** | **Stranding related comments** | **Nutritional condition** |
| --- | --- | --- | --- | --- | --- |
| M29/05 | Feb 2005 | Fraserburgh Beach, Aberdeenshire, Scotland | Adult female | Live stranded and euthanized on the basis of low body weight, hypothermia and possible gastric haemorrhage | Moderate |
| M21/09 | March 2009 | Balmedie, Aberdeenshire, Scotland | Adult female | Found dead | Moderate |
| M78/10 | April 2010 | Ettrick Bay, Isle of Bute, Scotland | Adult female | Found dead | Poor |
| UT692 | Feb 2012 | Oostende, Belgium | Adult female | Live stranded, transported to rehabilitation facility in the Netherlands, euthanized 4days post stranding on the basis of extensive loss of body weight and poor prognosis for recovery due to multiple external abscesses | Poor |
| SW12/463 | Oct 2012 | North Denes, Great Yarmouth, Norfolk, England | Adult female | Found dead | Poor |
| UT1576 | April 2017 | Egmond aan zee, the Netherlands | Juvenile female | Found dead | Poor |
| SW17/193 | April 2017 | Leysdown, Kent, England | Juvenile male | Found dead | Moderate to poor |
| M175/18 | March 2018 | Thorntonloch, near Dunbar, East Lothian, Scotland. | Adult female (pregnant) | Found dead | Poor |

**Table S2** Ancillary pathology findings per case which were not directly related to the *Neisseria* infection

| **Case ID** | **Ancillary pathology findings per case, not (in)directly related to *Neisseria* infection** | | | | |
| --- | --- | --- | --- | --- | --- |
|  | **Respiratory tract** | **Gastrointestinal tract** | **Liver** | **Other tissues** |  |
| M29/05 | Trachea, bronchi, lung parenchyma: presence of marked numbers of *Pseudalius inflexus* | Cardiac stomach: moderate mucosal ulceration with intralesional *Anisakis simplex* | Bile ducts: thickened and hyperplastic, associated with *Campula oblonga* infection | No ancillary abnormalities detected |  |
| M21/09 | Trachea, bronchi: small numbers of lung nematodes presence | No ancillary abnormalities detected | Bile ducts: thickened and hyperplastic, associated with *Campula oblonga* infection | Spleen: megakaryocytes present associated with extra-medullary haematopoiesis. |  |
| M78/10 | Trachea, bronchi, lung parenchyma: presence of nematodes | No ancillary abnormalities detected | Bile ducts: thickened and hyperplastic, mild, associated with *Campula oblonga* infection | Spleen and liver: megakaryocytes present associated with extra-medullary haematopoiesis. Auditory cavities: light burden of nematode parasites (*Stenurus minor*). |  |
| UT692 | Trachea, bronchi, lung parenchyma: presence of moderate numbers of nematodes (*Pseudalius inflexus* and *Torynurus convolutus*) | No ancillary abnormalities detected | No ancillary abnormalities detected | Urinary bladder: mild focal chronic lymphoplasmacytic cystitis. Auditory cavities: light burden of nematode parasites (*Stenurus minor*). |  |
| SW12/463 | Trachea, bronchi: lung nematodes present | No ancillary abnormalities detected | Bile ducts: thickened and hyperplastic, associated with *Campula oblonga* infection | No ancillary abnormalities detected |  |
| UT1576 | Trachea, bronchi, lung parenchyma: presence of severe numbers of nematodes (*Pseudalius inflexus* and *Torynurus convolutus*) | Stomach: small number of *Anisakis simplex* nematodes present. In stomach wall, small number of trematodes present (*Pholeter gastrophilus*). | No ancillary abnormalities detected | Auditory cavities: severe burden of nematode parasites (*Stenurus minor*). |  |
| SW17/193 | No ancillary abnormalities detected | Stomach: mucosal ulceration with small numbers of intralesional *Anisakis simplex* nematodes. In stomach wall small numbers of trematodes present (*Pholeter gastrophilus*). | No ancillary abnormalities detected | Auditory cavities: light burden of nematode parasites (*Stenurus minor*). |  |
| M175/18 | Trachea, bronchi, lung parenchyma: presence of moderate numbers of nematodes | No ancillary abnormalities detected | Bile ducts: mild *Campula oblonga* infection | No ancillary abnormalities detected |  |

**Table S3** Percentage cellular fatty acid composition of 3 isolates of *N. animaloris* compared with previously published isolates^3^

| **Fatty acid** | **M29/05** | **M21/09** | **M78/10** | **CFA Range for previously published isolates*** |
| --- | --- | --- | --- | --- |
| C_12 : 0_ | 8 | 8.4 | 8 | 6.4 – 8.7 |
| C_12 : 0 3-OH_ | 4.6 | 4.9 | 5 | 4.5 - 7.9 |
| C_14 : 0_ | 7.6 | 10 | 7.2 | 4.4 – 6.8 |
| C_16:1 w5c_ | 1.3 | 1.2 | 1.6 | 2 – 2.3 |
| C_16:1 w7c_ | 25.3 | 25.9 | 30.5 | 32.1 – 35.9 |
| C_16 : 0_ | 23.9 | 24 | 21 | 12.5 – 17.6 |
| C_18 : 1ω7c_ | 19.9 | 16.5 | 20.3 | 21.9 – 27.3 |
| C_18:0_ | Tr** | Tr | Tr | Tr |
| Summed feature 2*** | 4.9 | 5 | 3.5 | 3.1 – 5 |

* Data from^3^

**Tr, Trace amounts <1%

*** Summed feature 2 comprises C_14:0 3-OH_, C_16:1 iso_, an unidentified fatty acid with equivalent chain-length value of 10.928 or C_12_: ALDE, or any combination of these fatty acids.

**Table S4** Full bacteriology results of all cases

| **Case ID** | **Recovery sites**  ***N. animaloris*** | | **Other isolates** | | |
| --- | --- | --- | --- | --- | --- |
|  | **Location** | **growth** | **Species** | **Location** | **growth** |
| M29/05 | Lung abscess | profuse | *Salmonella enterica* 4,12:a:- | lung abscess | moderate |
|  | inguinal abscesses | profuse |  | Lung | moderate |
|  | tailstock lesions | profuse |  | Liver | light |
|  | Pus | profuse |  | Spleen | light |
|  |  |  |  | Kidney | light |
|  |  |  |  | mesenteric lymph node | light |
|  |  |  |  | intestine | light |
|  |  |  | *Corynebacterium sp.* | Brain | sparse |
| M21/09 | lung abscess | profuse | *Salmonella enterica* 4,12:a:- | lung abscess | light |
|  | Liver | scant |  | Kidney | light |
|  | Spleen | scant | *Actinobacillus delphinicola* | intestine | heavy mixed |
|  | Kidney | scant | coliform type organisms | intestine | heavy mixed |
|  | Brain | scant |  |  |  |
|  | intestine | scant |  |  |  |
| M78/10 | pectoral fin abscess | moderate | *Escherichia coli* | intestine | profuse |
|  | Lung abscess | moderate |  | Liver | light |
|  |  |  | Pasteurellaceae-like organism | uterine body | profuse |
| UT692 | shoulder abscess | light |  |  |  |
|  | pectoral fin abscess | light |  |  |  |
|  | lung abscess | light |  |  |  |
| SW12/463 | lung abscess | profuse | *Myroides* sp. | Lung | moderate |
|  | pectoral fin abscess | profuse |  | Brain | moderate |
|  | liver | profuse | *Enterococcus* sp. | Lung | moderate |
|  | kidney | profuse |  | Brain | moderate |
|  | milk | profuse | *Proteus mirabilis* | Lung abscess | profuse |
|  | pre scapular ln | profuse |  | Brain | profuse |
| UT1576 | lung abscess | profuse |  |  |  |
| SW17/193 | Lung abscess | moderate | *Staphylococcus* sp. | Kidney | moderate |
|  | invertebral tail stock abscess | moderate |  | tympanic bulla | moderate |
|  | left tail stock abscess | moderate | *Escherichia coli* | tympanic bulla | moderate |
|  | brain | moderate |  |  |  |
| M175/18 | lung abscess | profuse | *Brucella ceti* | lung 2 | sparse |
|  | lung 1 | moderate |  | Brain | light |
|  | lung 2 | moderate |  | placenta | light |
|  | pectoral fin lesion | moderate |  | pectoral fin lesion | sparse |
|  | pectoral fin bone | sparse |  | pectoral fin bone | light |
|  | pre-scapular lymph node | profuse | *Escherichia coli* | pectoral fin lesion | profuse |
|  |  |  | *Enterococcus* sp. | pectoral fin lesion | moderate |
|  |  |  | *Proteus* | pectoral fin lesion | light |
